# Comparative Effectiveness, Safety, and Cost-Effectiveness of Labour Induction Methods: A Systematic Review

**DOI:** 10.7759/cureus.93382

**Published:** 2025-09-27

**Authors:** Shahinaz Abdelgaium Elsamani Mohamed, Mohamed Hamid El Hassan Hamid

**Affiliations:** 1 Obstetrics and Gynaecology, Royal Surrey County Hospital, Guildford, GBR; 2 Obstetrics and Gynaecology, Dorset County Hospital NHS Foundation Trust, Dorchester, GBR

**Keywords:** cervical ripening, cost-effectiveness, foley catheter, labour induction, misoprostol, obstetrics, randomized controlled trials, systematic review

## Abstract

Labour induction is a common obstetric intervention, most often undertaken at term in women with an unfavourable cervix, and its rising global use underscores the need to identify optimal strategies that balance effectiveness, safety, and cost-effectiveness. This systematic review aimed to evaluate the comparative effectiveness, safety, and cost-effectiveness of pharmacological, mechanical, and combined methods of induction in term pregnancies, including both nulliparous and multiparous women undergoing elective or indicated induction. The review followed the Preferred Reporting Items for Systematic Reviews and Meta-Analyses (PRISMA) guidelines. A systematic search of PubMed, Scopus, Web of Science, and Embase was performed for randomized controlled trials (RCTs) published between 2015 and 2025. Ten RCTs were included and assessed for risk of bias using the Cochrane RoB 2 tool. A narrative synthesis was performed due to clinical and methodological heterogeneity. Across 10 RCTs (n=8,683 participants), oral misoprostol and Foley catheter demonstrated comparable effectiveness and safety for cervical ripening and induction. Combination methods (e.g., misoprostol plus Foley catheter) significantly shortened induction-to-delivery intervals and increased the likelihood of vaginal birth within 24 hours compared with single methods. Safety outcomes were generally favourable, although higher rates of infectious morbidity were reported with mechanical methods in women with prelabour rupture of membranes. Cost analyses indicated that misoprostol and Foley catheter incur similar inpatient costs, while outpatient Foley catheter ripening provides substantial savings. Additionally, induction at 41 weeks was more cost-effective than expectant management until 42 weeks, particularly for nulliparous women. The evidence suggests that oral misoprostol and Foley catheter are equally effective first-line options, while combination methods and outpatient strategies may optimize outcomes depending on parity, cervical status, and healthcare setting.

## Introduction and background

Labour induction is one of the most frequently performed obstetric interventions worldwide, undertaken when the benefits of expediting delivery outweigh the risks of continuing pregnancy [1,2]. Induction rates have risen steadily over the past two decades, with an estimated 29% of all births in high-income countries and an increasing trend also being observed in low- and middle-income settings [3]. This growing prevalence underscores the importance of identifying induction strategies that are not only clinically effective but also safe and cost-efficient, given their broad impact on maternal and neonatal outcomes as well as healthcare systems [4].

Multiple pharmacological, mechanical, and combined methods have been developed and implemented for induction, including prostaglandins, oxytocin, misoprostol, Foley catheter, and combination approaches such as prostaglandins or misoprostol used together with a Foley catheter [5]. Considerable evidence exists regarding the efficacy of these individual methods, but their relative benefits remain uncertain. While some trials suggest comparable success rates, others highlight differences in vaginal delivery within 24 hours, caesarean section rates, and risks of uterine hyperstimulation or adverse neonatal outcomes [6]. These inconsistencies reflect variation in study design, populations, and clinical settings, leaving important questions unanswered about which strategies are most effective and safest in specific contexts [7]. Furthermore, induction choices are often shaped by local guidelines, resource availability, and patient-specific factors such as parity, gestational age, and comorbidities [8].

In addition, the economic implications of induction methods are increasingly relevant. Rising healthcare costs and limited resources, particularly in resource-constrained settings, demand the careful evaluation of cost-effectiveness alongside clinical outcomes. Although some methods may reduce hospital stay or operative delivery, evidence on their economic impact remains fragmented and context-dependent.

Given these uncertainties, a systematic synthesis of current evidence is needed to clarify the comparative effectiveness, safety, and cost-effectiveness of available methods. Previous reviews have provided valuable insights [9-11], but they are limited by rapid changes in obstetric practice, the emergence of combination strategies, and growing emphasis on cost-effectiveness. An updated and comprehensive assessment is therefore necessary to inform clinical decision-making and guide health policy development.

## Review

Methodology

Protocol and Registration

This systematic review was conducted in accordance with the Preferred Reporting Items for Systematic Reviews and Meta-Analyses (PRISMA) 2020 guidelines [12]. The review protocol was developed prospectively to ensure transparency and methodological rigor. Although the protocol was not registered in the International Prospective Register of Systematic Reviews (PROSPERO) due to administrative limitations, all methodological decisions were made prior to data extraction to minimize bias and enhance reproducibility.

Eligibility Criteria

Studies were included if they were randomized controlled trials (RCTs) published between January 2015 and January 2025 that evaluated pharmacological (e.g., prostaglandins, oxytocin, misoprostol), mechanical (e.g., Foley catheter, double-balloon catheter), or combination methods for labour induction. We excluded non-randomized studies, case series, narrative reports, conference abstracts without full data, non-English publications, studies not reporting relevant maternal or neonatal outcomes, and those assessing experimental or obsolete techniques to ensure methodological rigor and contemporary clinical relevance (Table 1).

**Table 1:** Eligibility criteria

Criterion	Inclusion	Exclusion
Study design	Randomized controlled trials	Observational studies, cohort studies, case-control studies, case reports, systematic/narrative reviews
Population	Pregnant women undergoing labour induction	Animal studies, non-pregnant populations
Interventions	Any labour induction method (pharmacological, mechanical, combined)	Non-induction interventions (e.g., elective caesarean, expectant management)
Comparators	Other induction methods, placebo, or standard care	Studies without a comparator
Outcomes	Effectiveness (time to delivery, vaginal delivery rates), safety (maternal and neonatal outcomes), cost-effectiveness	Studies not reporting relevant outcomes
Publication date	2015-2025	Prior to 2015
Language	English	Non-English studies

Information Sources

A comprehensive literature search was performed across four major electronic databases: PubMed, Scopus, Web of Science, and Embase. These databases were selected to ensure broad coverage of biomedical, clinical, and interdisciplinary literature. The last search was conducted in July 2025. To supplement the database search, reference lists of included studies and relevant systematic reviews were screened to identify additional eligible articles.

Search Strategy

The search strategy was developed using a combination of Medical Subject Headings (MeSH) and free-text terms related to "labour induction", "induction of labor", "pharmacological agents", "mechanical methods", "safety", "effectiveness", and "cost-effectiveness". Boolean operators ("AND", "OR") were used to combine terms appropriately. Filters for study design (RCTs), publication date (2015-2025), and language (English) were applied (Table 2).

**Table 2:** Example of PubMed search strategy MeSH: Medical Subject Headings; RCTs: randomized controlled trials

Search concept	Keywords/MeSH terms
Population	“pregnancy”[MeSH] OR “pregnant women” OR “labor, obstetric”[MeSH]
Intervention	“labor induction”[MeSH] OR “induction of labor” OR “cervical ripening”
Comparators	“prostaglandins” OR “oxytocin” OR “misoprostol” OR “Foley catheter” OR “mechanical methods”
Outcomes	“effectiveness” OR “safety” OR “cost-effectiveness” OR “health economics”
Filters	Publication date: 2015-2025. Study design: RCTs. Language: English

The search strategies were adapted to suit the indexing systems of Scopus, Web of Science, and Embase.

Selection Process

All retrieved records were imported into EndNote X9 (Clarivate, London, United Kingdom) to facilitate reference management and deduplication. Titles and abstracts were screened independently by two reviewers against eligibility criteria. Full-text screening was performed for potentially eligible articles. Discrepancies were resolved through discussion or consultation with a third reviewer to ensure consistency and minimize selection bias.

Data Collection Process

Data extraction was carried out independently by two reviewers using a standardized extraction form. Extracted information included study characteristics (author, year, country, population), induction methods, comparators, primary and secondary outcomes, and follow-up duration. Any disagreements were resolved by consensus.

Data Items

Primary outcomes included effectiveness outcomes (time to delivery, vaginal delivery rates, caesarean section rates), safety outcomes (maternal morbidity, postpartum hemorrhage, neonatal outcomes such as Apgar score and neonatal intensive care unit (NICU) admission), and cost-effectiveness outcomes (direct and indirect healthcare costs, hospital stay duration).

Study Risk of Bias Assessment

The Cochrane RoB 2 tool was used to evaluate methodological quality [13]. Each included study was assessed across five domains: bias arising from the randomization process, deviations from intended interventions, missing outcome data, measurement of the outcome, and selection of the reported result. Two reviewers independently assessed risk of bias, and disagreements were resolved through discussion.

Effect Measures

Given the diversity of outcome reporting across trials, risk ratios (RR), odds ratios (OR), and mean differences (MD) were considered for effectiveness and safety outcomes, while cost-effectiveness was summarized narratively.

Synthesis Methods

Due to substantial clinical heterogeneity across included trials in terms of interventions, comparators, outcome definitions, and cost-analysis methodologies, a meta-analysis was not conducted. Pooling such heterogeneous data would risk producing misleading or non-generalizable results. Instead, a narrative synthesis was undertaken, structured around the type of induction method, outcome category, and healthcare context.

Reporting Bias Assessment

Efforts to minimize publication bias included a broad database search, screening of reference lists, and inclusion of unpublished trial data when available. However, formal statistical assessment of reporting bias (e.g., funnel plot analysis) was not feasible given the absence of meta-analysis.

Results

Study Selection Process

**Figure 1:**
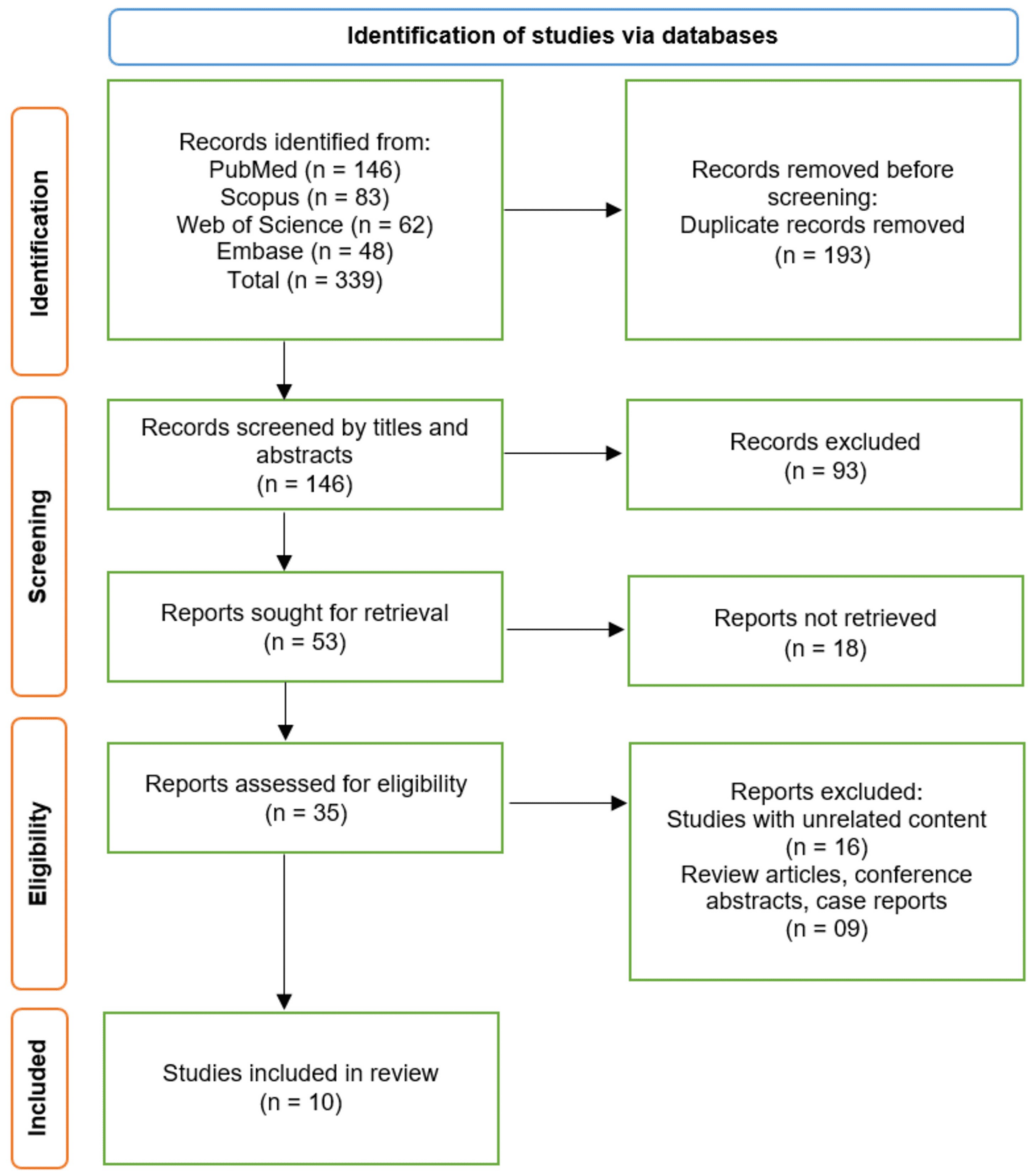
Study selection process illustrated on the PRISMA flowchart PRISMA: Preferred Reporting Items for Systematic Reviews and Meta-Analyses

The study selection process, detailed according to the PRISMA flow diagram, is summarized in Figure 1. A systematic search of four electronic databases (PubMed, Scopus, Web of Science, and Embase) was conducted, initially identifying 339 records. After the removal of 193 duplicate records, 146 unique studies were screened based on their titles and abstracts. This screening phase led to the exclusion of 93 records that did not meet the inclusion criteria. Subsequently, the full texts of the remaining 53 reports were sought for retrieval; of these, 18 could not be retrieved. Therefore, 35 full-text articles were assessed for eligibility. Upon detailed evaluation, 25 articles were excluded for the following reasons: 16 studies had unrelated content, and nine were review articles, conference abstracts, or case reports. Ultimately, 10 studies met all the pre-defined eligibility criteria and were included in the qualitative synthesis of this systematic review [14-23].

Characteristics of the Included Studies

A total of 10 studies were included in this systematic review, the characteristics of which are summarized in Table 3 [14-23]. The studies were published between 2015 and 2025, with sample sizes ranging from 175 to 1,859 participants. Geographically, the studies were conducted across the Netherlands [14,15,19], China [16], India [17], the United States [18], Ireland [20], Finland [21,22], and Iran [23]. All studies were RCTs, with four designed as non-inferiority trials [14,20-22]. The populations consisted of women with term (≥37-week) pregnancies, with some studies focusing on specific subgroups such as those with an unfavourable cervix [14-18,23], late-term (41-42-week) pregnancies [19,22], nulliparous women [22], or women with prelabour rupture of membranes (PROM) [23].

**Table 3:** Characteristics of the included studies RCT: randomized controlled trial; IOL: induction of labour; EM: expectant management; NR: not reported; PROM: prelabour rupture of membranes; PPH: postpartum hemorrhage; CAPO: composite adverse perinatal outcome; NICU: neonatal intensive care unit; SAPO: severe adverse perinatal outcome; UA: umbilical artery

Author, year	Country	Study design	Population (n)	Gestational age (weeks)	Induction method(s)	Comparator(s)	Primary outcomes measured	Follow-up duration
Ten Eikelder et al. (2016) [14]	Netherlands	Open-label randomized non-inferiority trial	1,859 (932 misoprostol; 927 Foley)	Term (≥37 weeks)	Oral misoprostol (50 μg every 4 h)	Foley catheter (30 mL transcervical)	Composite of neonatal asphyxia (pH ≤7.05 or 5-min Apgar <7) and postpartum hemorrhage (≥1000 mL); caesarean section rate; adverse events	Until delivery and immediate postpartum outcomes
Ten Eikelder et al. (2018) [15]	Netherlands	RCT with economic evaluation	1,845 (misoprostol: 924; Foley: 921)	Term (≥37 weeks, viable singleton)	Oral misoprostol	Foley catheter	Direct medical costs associated with healthcare utilization	From randomization until hospital discharge
Gu et al. (2015) [16]	China	Open-label RCT	504 women (126 in each group; 9 excluded post-randomization)	Term (≥37 weeks)	Foley catheter balloon (30 mL for 12 h, 30 mL for 24 h, 80 mL for 12 h, 80 mL for 24 h)	Different balloon volumes (30 mL vs. 80 mL) and ripening durations (12 h vs. 24 h)	Vaginal delivery within 24 hours (primary); caesarean section rate; maternal/neonatal morbidity (secondary)	Until delivery (per-protocol basis)
Anjali et al. (2022) [17]	India	RCT	200	Term gestation	Oral misoprostol (25 µg every 2 h) + Foley catheter	Oral misoprostol alone (25 µg every 2 h)	Induction-to-delivery interval, vaginal delivery within 24 h, maternal adverse events, neonatal outcomes (Apgar score, PPH)	Until delivery (intrapartum and immediate neonatal outcomes)
Pimentel et al. (2018) [18]	USA	RCT	250 women	≥37 weeks	Single-dose misoprostol	Multiple-dose misoprostol (up to 4 doses)	Vaginal delivery within 24 hours (primary). Secondary: time to vaginal delivery, caesarean delivery rate, maternal and neonatal morbidity	Until delivery (peripartum follow-up)
Bruinsma et al.(2023) [19]	Netherlands	RCT	1801 low-risk women (IOL: 900, EM: 901)	41-42 weeks (late-term pregnancy)	Elective IOL at 41 weeks	EM until 42 weeks	Direct medical costs, CAPO (perinatal mortality, NICU admission, Apgar <7 at 5 min, plexus brachialis injury, meconium aspiration), composite SAPO (Apgar <4 at 5 min, etc.)	Until delivery and perinatal outcome assessment
Nicholson et al. (2024) [20]	Ireland	Phase III, open-label, single-center, non-inferiority RCT	327 randomized; 271 completed	39 weeks (elective IOL)	Dilapan-S for 12 h (D12), Dilapan-S for 24 h (D24), Propess for 24 h (P24)	Dilapan-S (D12/D24) vs. Propess (P24)	Vaginal delivery at any time (primary); secondary: time to delivery, need for additional ripening, hospital stay, adverse events	Up to 72 h from the initiation of induction process
Kruit and Rahkonen (2025) [21]	Finland (Helsinki University Hospital)	RCT	175 women with PROM	Term (≈37-42 weeks, implied for PROM at term)	Balloon catheter ± prophylactic antibiotics	Low-dose oral misoprostol (25 µg)	Mode of delivery, maternal infection (chorioamnionitis), neonatal infection, maternal childbirth satisfaction	In-hospital period until delivery and immediate postpartum outcomes
Kruit et al. (2025) [22]	Finland	Randomized multicenter pilot trial	273 nulliparous women	41+0 weeks (late- and post-term)	Balloon catheter, oral misoprostol (50 μg q4h), combination of both	Balloon catheter vs. oral misoprostol vs. combination	Caesarean section rate. Composite adverse neonatal outcomes (5-min Apgar <7, UA pH ≤7.05, NICU admission). Induction-to-delivery interval	Until delivery and immediate neonatal outcomes
Beyrami et al. (2024) [23]	Iran (Tehran)	Randomized, open-label clinical trial	NR	Groups homogeneous (p=0.44)	Oral misoprostol + Foley catheter	Oral misoprostol alone	Bishop score at baseline, 6 h, and 12 h; number of deliveries within 24 h; oxytocin dosage; maternal complications (e.g., tachysystole); fetal outcomes (Apgar score, NICU admission, meconium presence)	Until delivery (outcomes assessed within 24 h and immediate neonatal period)

The primary induction methods investigated included pharmacological agents (oral misoprostol at varying doses and intervals [14,15,17,18,21-23]), mechanical methods (Foley catheter [14-17,22,23] or Dilapan-S [20]), and a combination of both [17,22,23]. Comparators were either another active method (e.g., Foley catheter vs. misoprostol) or expectant management (EM) [19]. The primary outcomes were diverse, encompassing efficacy endpoints such as vaginal delivery within 24 hours [16-18,20,23], caesarean section rate [14,16,22], time intervals from induction to delivery [17,18,22], and safety composites for maternal and neonatal morbidity [14,19]. Follow-up is typically extended through delivery and the immediate postpartum period.

Synthesis of Effectiveness Outcomes

The effectiveness of various induction methods, particularly regarding the rate of vaginal delivery and time to delivery, was a primary focus across all studies. Oral misoprostol and Foley catheter were found to have comparable effectiveness. The large PROBAAT-II trial [14] found no significant difference in vaginal birth rates or the rate of caesarean section (16.8% vs. 20.1%; RR 0.84; 95% CI 0.69-1.02; p=0.067) between the two methods.

Several studies indicated that combination methods could enhance efficacy. Anjali et al. [17] and Beyrami et al. [23] both reported that combining low-dose oral misoprostol or pitocin with a Foley catheter significantly shortened the induction-to-delivery interval and increased the proportion of women achieving vaginal delivery within 24 hours compared to misoprostol or pitocin alone. Similarly, Kruit et al. [22] found that a combination of a balloon catheter and oral misoprostol resulted in the shortest induction-to-delivery time (21.7 hours) and the highest rate of delivery within 24 hours (54.4%) compared to either method used alone.

The timing and dosage of methods also influenced outcomes. Gu et al. [16] demonstrated that a shorter 12-hour Foley catheter ripening duration was more effective for achieving vaginal delivery within 24 hours than a 24-hour duration, though balloon volume (30 mL vs. 80 mL) made no significant difference. In contrast, Pimentel et al. [18] found no significant difference in the rate of vaginal delivery within 24 hours or time to vaginal delivery between a single-dose and a multiple-dose misoprostol regimen. Regarding outpatient management, Nicholson et al. [20] reported that a 24-hour duration with either Dilapan-S or Propess achieved high vaginal delivery rates (75% and 76%, respectively), while a 12-hour Dilapan-S protocol was less effective (64%).

Synthesis of Safety Outcomes

The safety profiles of the induction methods were generally reassuring, with most studies reporting no significant differences in serious maternal or neonatal adverse events between comparison groups. The PROBAAT-II trial [14] reported a composite adverse outcome (neonatal asphyxia or postpartum hemorrhage) of 12.2% for misoprostol and 11.5% for Foley catheter, with no procedure-related serious adverse events.

Specific safety signals were noted in some studies. Anjali et al. [17] reported a higher incidence of postpartum hemorrhage and low five-minute Apgar scores in the misoprostol-only group compared to the combination group. In the context of PROM, Kruit and Rahkonen [21] observed a non-significant trend towards higher rates of maternal chorioamnionitis (9.1% vs. 3.5%) and neonatal infection (4.5% vs. 2.3%) with a balloon catheter compared to oral misoprostol, noting that antibiotic prophylaxis did not mitigate this risk. Conversely, Beyrami et al. [23] found that the combination of misoprostol and Foley catheter was associated with lower rates of tachysystole, NICU admission, low Apgar scores, and meconium presence than misoprostol alone.

For studies comparing induction of labour (IOL) to EM, Bruinsma et al. [19] evaluated composite adverse perinatal outcomes (composite adverse perinatal outcome (CAPO) and severe adverse perinatal outcome (SAPO)) and found that the choice between IOL at 41 weeks and EM until 42 weeks involved a trade-off between potential benefits and harms, with the cost-effectiveness analysis suggesting the decision may differ based on parity.

Synthesis of Cost-Effectiveness Outcomes

The cost-effectiveness of induction methods was directly evaluated in three studies [15,19,20], with others suggesting potential economic implications through reduced resource use [17,18,22]. The economic analysis of the PROBAAT-II trial by Ten Eikelder et al. [15] found that the mean cost per woman was comparable between oral misoprostol (€4470) and Foley catheter (€4158), with a non-significant mean difference of €312. However, a key finding was that outpatient cervical ripening with a Foley catheter could reduce costs to €3489, representing a potential saving of nearly €1000 per woman compared to misoprostol.

Bruinsma et al. [19] conducted a cost-effectiveness analysis of IOL at 41 weeks versus EM until 42 weeks. The mean cost was slightly higher for IOL (€3858 vs. €3723). The incremental cost-effectiveness ratio (ICER) was €9,436 per CAPO prevented. The cost-effectiveness acceptability curve (CEAC) showed an 80% probability that IOL at 41 weeks is cost-effective at willingness-to-pay thresholds of €22,000 (for CAPO) and €50,000 (for SAPO). This probability was much higher for nulliparous women than for multiparous women.

Although Nicholson et al. [20] did not provide a formal cost analysis, they highlighted that outpatient cervical ripening with devices like Dilapan-S or Propess has the potential for significant cost savings by reducing the duration of hospital stay and freeing up inpatient resources.

A summary of the effectiveness, safety, and cost-effectiveness findings from all included studies is presented in Table 4 [14-23].

**Table 4:** Effectiveness, safety, and cost-effectiveness of labour induction methods IOL: induction of labour; EM: expectant management; CAPO: composite adverse perinatal outcome; NICU: neonatal intensive care unit; SAPO: severe adverse perinatal outcome; ICER: incremental cost-effectiveness ratio; CEAC: cost-effectiveness acceptability curve; BC: balloon catheter; OM: oral misoprostol

Author, year	Effectiveness outcomes	Safety outcomes	Cost-effectiveness outcomes	Key findings
Ten Eikelder et al. (2016) [14]	Vaginal birth rates (comparable between groups). Caesarean section: 16.8% (misoprostol) vs. 20.1% (Foley); RR 0.84; 95% CI 0.69-1.02; p=0.067	Composite adverse outcome (asphyxia (pH ≤7.05 or 5-min Apgar <7) or postpartum hemorrhage ≥1000 mL): 12.2% (misoprostol) vs. 11.5% (Foley); ARR 1.06; 90% CI 0.86-1.31. Adverse events: 27 (misoprostol) vs. 25 (Foley); none directly related to procedure	No cost-effectiveness data provided	Oral misoprostol and Foley catheter showed similar effectiveness and safety for IOL in women with unfavourable cervix. Slight trend toward lower caesarean rates with misoprostol
Ten Eikelder et al. (2018) [15]	Trial compared oral misoprostol vs. Foley catheter for induction	No adverse events or maternal or neonatal complications reported	Mean costs: oral misoprostol €4470 vs. Foley catheter €4158 (mean difference €312; 95% CI -€508 to €1063). In sensitivity analysis, outpatient cervical ripening with Foley catheter reduced costs further to €3489, saving nearly €1000 per woman compared to misoprostol	Oral misoprostol and Foley catheter had comparable costs overall. Outpatient use of Foley catheter for low-risk women could lead to significant cost savings
Gu et al. (2015) [16]	Primary outcome: vaginal delivery within 24 hours was higher in the 12-hour Foley catheter groups compared to the 24-hour groups (30 mL/12 h: 54.5%; 30 mL/24 h: 33.1%; 80 mL/12 h: 46.4%; 80 mL/24 h: 24%; p<0.001). Secondary outcome: caesarean section rates were comparable among groups	Incidence of chorioamnionitis and maternal/neonatal morbidity were comparable among all four groups	Not reported	Foley catheter is safe and effective for IOL in women with unfavourable cervix at term. Shorter ripening time (12 h) was more effective than 24 h, but balloon size (30 mL vs. 80 mL) did not significantly affect outcomes
Anjali et al. (2022) [17]	Shorter induction-to-active-labour interval in combination group (10.67±1.75 vs. 16.28±1.69 hours). Shorter induction-to-full-dilation interval (11.49 vs. 19.00 hours). Shorter induction-to-delivery interval (16.85 vs. 21.90 hours). Higher proportion of women delivering vaginally within 24 hours (76 vs. 57)	Postpartum hemorrhage significantly more frequent in oral misoprostol-only group; 5-minute Apgar score <7 more frequent in misoprostol-only group	Oral administration is convenient and may improve compliance, potentially lowering indirect costs compared to invasive methods	The combination of oral misoprostol with Foley catheter significantly reduced induction-to-delivery interval and increased the rate of vaginal delivery within 24 hours
Pimentel et al. (2018) [18]	Vaginal delivery within 24 hours: no significant difference between single-dose vs. multiple-dose misoprostol (41.7% vs. 44.7%; p=0.698). Time to vaginal delivery: no significant difference (1187 min vs. 1321 min; p=0.202)	Caesarean delivery rate higher in the single-dose group (35.8% vs. 22.8%; p=0.034), but association lost after adjusting for confounders	Findings suggest that a single-dose regimen may reduce medication use and hospital resource utilization if proven equally effective	Both regimens showed similar effectiveness in achieving vaginal delivery within 24 hours. The single-dose group initially showed higher caesarean rate, but adjusted analysis indicated that parity and Bishop score, not dosage regimen, were the significant predictors
Bruinsma et al.(2023) [19]	Compared IOL at 41 weeks with EM until 42 weeks	CAPO: perinatal mortality, NICU admission, Apgar 5 min <7, plexus brachialis injury, and/or meconium aspiration syndrome. Composite SAPO: included Apgar 5 min <4 instead of <7	Mean costs: €3858 (IOL) vs. €3723 (EM); mean difference €135 (95% CI -235 to 493). ICER: €9436 per CAPO prevented and €14,994 per SAPO prevented. CEAC: 80% probability of cost-effectiveness at willingness-to-pay €22,000 (CAPO) and €50,000 (SAPO). Subgroup (nulliparous): willingness-to-pay €47,000 (CAPO) and €62,000 (SAPO). Subgroup (multiparous): willingness-to-pay €190,000 (CAPO) and €970,000 (SAPO)	Induction at 41 weeks has an 80% probability of being cost-effective at €22,000 (CAPO) and €50,000 (SAPO). More likely cost-effective for nulliparous women, but unlikely for multiparous women. Cost-effectiveness may vary across health systems and populations
Nicholson et al. (2024) [20]	Primary outcome: vaginal delivery rates (75% in D24, 76% in P24, and 64% in D12). Secondary outcomes: time to delivery (majority delivered within 72 h across groups: 89% D24, 98% D12, and 95% P24)	No significant differences in maternal or neonatal adverse events across groups	Outpatient cervical ripening with Dilapan-S (D24) or Propess (P24) potentially reduces hospital stay time by allowing part of the induction process at home. This may translate into improved resource use and cost savings, although cost data was not directly measured	D24 and P24 achieved similarly high vaginal delivery rates (≥75%). D12 had lower vaginal delivery rate (64%) and failed to demonstrate non-inferiority compared with Propess. Outpatient cervical ripening was safe, effective, and logistically feasible, with potential cost benefits through reduced inpatient time
Kruit and Rahkonen (2025) [21]	Mode of delivery (caesarean section rates: BC 19.1% vs. OM 11.6%; p=0.17)	Maternal infections: chorioamnionitis (BC 9.1% vs. OM 3.5%; p=0.21). Neonatal infections (BC 4.5% vs. OM 2.3%; p=0.68)	Not assessed in this study	Caesarean delivery rates were comparable between BC and OM. There was a non-significant trend toward higher maternal and neonatal infections with BC compared to OM. Routine antibiotic prophylaxis during BC did not reduce infection incidence
Kruit et al. (2025) [22]	Caesarean section rates similar (23.7% balloon, 24.5% misoprostol, 17.1% combo). Shortest induction-to-delivery time with combo (21.7 h). More deliveries ≤24 h in combo (54.4%)	Neonatal adverse outcomes similar (≈7% each). No safety concerns	Shorter time may reduce resource use	Combo method more effective (faster delivery). Safety comparable. May offer efficiency benefits
Beyrami et al. (2024) [23]	Higher Bishop scores at 6 and 12 h; more deliveries within 24 h (failure: 11.6% vs. 73.4%)	Lower tachysystole, NICU admission, low Apgar, and meconium	Not reported	Combo (misoprostol + Foley) more effective and safer than misoprostol alone

Risk of Bias Findings

**Figure 2:**
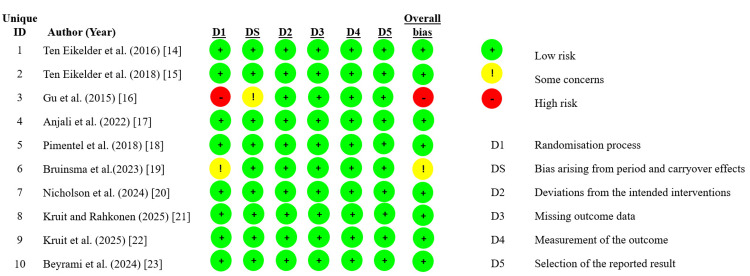
Heatmap of risk of bias assessment on Cochrane RoB 2 tool

The risk of bias assessment using the RoB 2 tool showed that most studies, including Ten Eikelder et al. [14,15], Anjali et al. [17], Pimentel et al. [18], Nicholson et al. [20], Kruit and Rahkonen [21], Kruit et al. [22], and Beyrami et al. [23], were judged to have a low risk of bias across all domains. The study by Gu et al. [3] was assessed as having a high risk of bias due to issues in the randomization process and period/carryover effects, while that of Bruinsma et al. [19] was rated as having some concerns, primarily related to the randomization process. Overall, the majority of studies were of low risk, with only one study at high risk [16] and one study with some concerns [19] (Figure 2).

Discussion

This systematic review synthesizes evidence from 10 RCTs to evaluate the comparative effectiveness, safety, and cost-effectiveness of methods for labour induction at term. The findings highlight that, due to substantial heterogeneity across studies, the optimal induction strategy depends on a combination of clinical context, patient characteristics, and healthcare system considerations rather than a single universally superior method. The foundational conclusion, robustly supported by the large PROBAAT-II trial, is that oral misoprostol and Foley catheter are fundamentally equivalent in their overall ability to achieve vaginal birth, with no statistically significant difference in caesarean section rates or serious composite adverse outcomes [14]. This equivalence is a critical starting point, as it liberates clinical decision-making from a rigid hierarchy of methods and allows for choices to be tailored to specific circumstances. However, this review delves deeper, demonstrating that strategic enhancements, particularly through combination therapy, can improve upon these baseline outcomes. The consistent findings of Anjali et al. and Beyrami et al. that combining low-dose oral misoprostol with a Foley catheter significantly shortens the induction-to-delivery interval and increases the rate of vaginal delivery within 24 hours suggest a powerful synergistic effect [17,23]. This synergy is biologically coherent; the mechanical dilation provided by the Foley catheter likely causes local prostaglandin release and physical ripening, which primes the cervix to be more responsive to the exogenous prostaglandin administered orally, thereby creating a more efficient and physiological induction process. This is further corroborated by Kruit et al., who found the combination to be superior to either method alone in nulliparous women at late term, a group known for having particularly unfavourable cervices and a higher risk of prolonged induction and caesarean delivery [22]. This suggests that for these higher-risk inductions, a combination approach may be the most effective strategy to optimize outcomes.

The review also highlights the importance of protocol specifics, where the devil is often in the details. The work of Gu et al. provides a seminal insight into mechanical ripening: duration matters more than volume [16]. Their finding that a 12-hour Foley catheter ripening period was significantly more effective than a 24-hour period challenges traditional, more passive management and indicates that there is an optimal window for mechanical stimulation beyond which additional time may not yield benefit and could potentially increase discomfort and infection risk. In contrast, the pharmacological dosing regimen appears more flexible. Pimentel et al. found that a single dose of misoprostol was as effective as multiple doses, and their subsequent analysis revealing that parity and Bishop score were the true determinants of outcome is a crucial reminder that patient-specific factors often outweigh minor variations in intervention protocol [18]. This aligns with a large body of obstetric literature, such as the studies by Grobman et al. [24] in the ARRIVE trial, which demonstrated that patient factors are primary drivers of labour outcomes and interventions work within those constraints. Furthermore, the successful outpatient management protocols described by Nicholson et al. and the economic analysis of Ten Eikelder et al. underscore a significant shift in care paradigms [15,20]. Moving the initial ripening phase out of the inpatient setting is not only safe and effective for low-risk women but also represents a major opportunity for healthcare systems to improve efficiency and reduce costs, a priority in modern maternity care.

On the critical issue of safety, the overall narrative is reassuring for the general population, with the PROBAAT-II trial reporting equivalent and low rates of serious adverse events for both misoprostol and Foley catheter [14]. However, this review importantly identifies that safety is not absolute but context-dependent. The trend observed by Kruit and Rahkonen towards higher infectious morbidity with a balloon catheter in women with PROM is a vital safety signal [21]. In the context of broken membranes, the introduction of a foreign body into the cervical canal may facilitate ascending infection, a risk that is inherently avoided with a pharmacological agent. This finding suggests that in this specific subpopulation, oral misoprostol may be the safer first-line option, a recommendation that aligns with guidelines from the Royal College of Obstetricians and Gynaecologists [25]. Conversely, the improved safety profile of the combination method over misoprostol alone, as reported by Beyrami et al. [23], likely stems from the ability to use a lower dose of misoprostol to achieve efficacy, thereby reducing the risk of dose-related side effects like uterine tachysystole. This illustrates the complex risk-benefit calculus that clinicians must perform, where the safety of a method is intimately tied to the clinical scenario in which it is applied.

The incorporation of economic evaluations elevates this review from a purely clinical discussion to one that resonates with healthcare policymakers and administrators. The analysis by Ten Eikelder et al. demonstrates that the direct costs of the methods themselves are comparable, but the real financial leverage lies in care pathways [15]. The potential to save nearly €1000 per woman through outpatient Foley catheter ripening is a compelling argument for service reorganization. This is complemented by the findings of Bruinsma et al., which provide a sophisticated cost-effectiveness argument for when to induce, not just how to induce [19]. Their conclusion that induction at 41 weeks is likely cost-effective, but primarily for nulliparous women, is a landmark finding. It provides an economic rationale for tailoring policies based on parity, suggesting that a blanket policy for all women may not be an efficient use of resources. This nuanced approach to economic analysis moves beyond simple cost minimization to value-based care, where the expenditure is justified by the outcomes achieved in specific patient groups. This echoes the broader shift in healthcare towards precision medicine and personalized care pathways.

When contextualized within the wider literature, our findings both confirm and refine existing knowledge. The equivalence of misoprostol and Foley catheter is consistent with earlier network meta-analyses, such as the comprehensive work by Alfirevic et al., which concluded that both mechanical methods and low-dose misoprostol are effective with similar safety profiles [9,26]. Our review strengthens this evidence with more recent, high-quality trials. The superior efficacy of combination methods adds a new, impactful dimension to this evidence base, suggesting a strategy to improve upon the outcomes achieved by either method alone. The safety concern regarding Foley catheter use in PROM is a crucial nuance that must be integrated into clinical guidelines; while mechanical methods are generally safe, they may be contraindicated or used with extreme caution in specific high-risk situations. The economic evidence supporting outpatient Foley catheter ripening and induction at 41 weeks for nulliparous women provides a robust evidence base for policymakers and hospital administrators seeking to implement cost-effective practices that also align with patient preferences for minimizing hospital time where safe to do so. Our findings regarding the primacy of patient factors like parity are strongly supported by large cohort studies and trials like the ARRIVE trial, which established that underlying patient characteristics are often the most significant predictors of obstetric outcome [24].

Limitations

Despite its comprehensive nature, this systematic review has several limitations that must be acknowledged. Firstly, the included studies exhibited significant clinical and methodological heterogeneity. Populations varied, including general term pregnancies, nulliparous women, and those with PROM. Protocols for the same method (e.g., Foley catheter duration, misoprostol dose) also differed. This heterogeneity, while enriching the contextual analysis, precluded a formal meta-analysis, limiting our ability to provide pooled quantitative effect estimates. Secondly, the open-label design of most trials introduces a potential for performance bias, as clinicians and patients were not blinded to the intervention. This could have influenced decisions regarding co-interventions like oxytocin augmentation or caesarean section, though the use of objective primary outcomes likely mitigates this risk to some extent. Thirdly, the generalizability of findings may be limited. The studies were conducted in diverse healthcare settings across high- and middle-income countries. Protocols that are cost-effective and feasible in a well-resourced setting with established outpatient pathways may not be directly transferable to resource-constrained environments with different infrastructure and staffing models. Finally, the review primarily focused on clinical and economic outcomes. Patient-centered outcomes, such as satisfaction, experience of care, and perceived autonomy, were rarely reported but are increasingly recognized as critical components of high-quality maternity care.

## Conclusions

Oral misoprostol and Foley catheter are equally effective and safe for labour induction in the general obstetric population. However, it moves beyond this simple dichotomy to provide evidence for more nuanced and optimized approaches. The combination of mechanical and pharmacological methods appears to be particularly effective for accelerating labour, especially in nulliparous women with an unfavourable cervix. The safety profile of any method is context-dependent, with mechanical methods potentially carrying an increased risk of infection in women with PROM. Crucially, cost-effectiveness is influenced by the timing of induction, with evidence suggesting that induction at 41 weeks for nulliparous women may offer economic advantages without compromising safety. Therefore, the optimal method for labour induction is not singular but should be individualized, taking into account clinical context such as membrane status, patient characteristics like parity, and the resources available within the healthcare system. Future research should focus on standardizing outcome measures to facilitate meta-analysis, further exploring the optimal protocols for combination methods, and incorporating patient-centered outcomes into the evaluation of different induction strategies.
